# Islet autoantibody positivity in an adult population with recently diagnosed diabetes in Uganda

**DOI:** 10.1371/journal.pone.0268783

**Published:** 2022-05-23

**Authors:** Davis Kibirige, Isaac Sekitoleko, Priscilla Balungi, Jacqueline Kyosiimire-Lugemwa, William Lumu, Angus G. Jones, Andrew T. Hattersley, Liam Smeeth, Moffat J. Nyirenda

**Affiliations:** 1 Non-Communicable Diseases Program, Medical Research Council/Uganda Virus Research Institute and London School of Hygiene and Tropical Medicine Uganda Research Unit, Entebbe, Uganda; 2 Department of Non-Communicable Diseases Epidemiology, Faculty of Epidemiology and Population Health, London School of Hygiene and Tropical Medicine, London, United Kingdom; 3 Clinical Diagnostics Laboratory Services, Medical Research Council/Uganda Virus Research Institute, and London School of Hygiene and Tropical Medicine Uganda Research Unit, Entebbe, Uganda; 4 Department of Medicine, Mengo Hospital, Kampala, Uganda; 5 Institute of Biomedical and Clinical Science, University of Exeter Medical School, Barrack Road, Exeter, United Kingdom; 6 Department of Diabetes and Endocrinology, Royal Devon and Exeter NHS Foundation Trust, Exeter, United Kingdom; Sohag University Faculty of Medicine, EGYPT

## Abstract

**Aims:**

This study aimed to investigate the frequency of islet autoantibody positivity in adult patients with recently diagnosed diabetes in Uganda and its associated characteristics.

**Methods:**

Autoantibodies to glutamic acid decarboxylase-65 (GADA), zinc transporter 8 (ZnT8-A), and tyrosine phosphatase (IA-2A) were measured in 534 adult patients with recently diagnosed diabetes. Islet autoantibody positivity was defined based on diagnostic thresholds derived from a local adult population without diabetes. The socio-demographic, clinical, and metabolic characteristics of islet autoantibody-positive and negative participants were then compared. The differences in these characteristics were analysed using *the x*^2^ test for categorical data and the Kruskal Wallis test for continuous data. Multivariate analysis was performed to identify predictors of islet autoantibody positivity.

**Results:**

Thirty four (6.4%) participants were positive for ≥1 islet autoantibody. GADA, IA-2A and ZnT8-A positivity was detected in 17 (3.2%), 10 (1.9%), and 7 (1.3%) participants, respectively. Compared with those negative for islet autoantibodies, participants positive for islet autoantibodies were more likely to live in a rural area (n = 18, 52.9% Vs n = 127, 25.5%, p = 0.005), to be initiated on insulin therapy (n = 19, 55.9% Vs n = 134, 26.8%, p<0.001), to have a lower median waist circumference (90 [80–99] cm Vs 96 [87–104.8], p = 0.04), waist circumference: height ratio (0.55 [0.50–0.63] vs 0.59 [0.53–0.65], p = 0.03), and fasting C-peptide concentration (0.9 [0.6–1.8] Vs 1.4 [0.8–2.1] ng/ml, p = 0.01). On multivariate analysis, living in a rural area (odds ratio or OR 3.62, 95%CI 1.68–7.80, p = 0.001) and being initiated on insulin therapy (OR 3.61, 95% CI 1.67–7.83, p = 0.001) were associated with islet autoantibody positivity.

**Conclusion:**

The prevalence of islet autoantibody positivity was relatively low, suggesting that pancreatic autoimmunity is a rare cause of new-onset diabetes in this adult Ugandan population. Living in a rural area and being initiated on insulin therapy were independently associated with islet autoantibody positivity in this study population.

## Introduction

Autoimmune diabetes in European and Asian populations occurs throughout life with at least half of it occurring in adults [1–4]. Islet autoantibodies as markers of beta-cell autoimmunity may be detected in adults with clinically presumed new-onset type 2 diabetes. This category of patients is often described as latent autoimmune diabetes in adults (LADA) and possesses phenotypic features that are difficult to distinguish from type 2 diabetes at diagnosis in absence of pancreatic autoantibody testing [5, 6]. It is important to note that a proportion of these LADA cases may represent false-positive test results given the imperfect specificity of the islet autoantibody assays [6–8].

In resource-constrained settings like sub-Saharan Africa (SSA), screening for common islet autoantibodies like antibodies to glutamic acid decarboxylase-65 (GADA), zinc transporter 8 (ZnT8-A), tyrosine phosphatase (IA-2A), and insulin (IAA) is not feasible in routine clinical practice. Diagnosis of type 1 diabetes is usually based on the presence of distinctive clinical features like low body mass index (BMI) and age at diagnosis, ketosis, and other features of insulin deficiency. This poses a major diagnostic challenge because SSA is known to harbour atypical diabetes phenotypes that manifest with similar clinical features, such as the presence of type 2 diabetes in relatively lean individuals, fibro calculous pancreatic diabetes, and ketosis-prone diabetes [9, 10].

The true prevalence and features associated with islet autoantibody positivity in adult patients with clinically presumed new-onset type 2 diabetes in SSA are largely unknown. Most studies have enrolled individuals with long-standing diabetes, which can influence the frequency and pattern of islet autoantibodies detected [11–20]. Indeed, in most cases, only one islet autoantibody (commonly GADA) has been measured, with the potential to underestimate the prevalence of pancreatic autoimmunity. Conversely, in most studies, islet autoantibody positivity has been defined based on manufacturer’s cut-offs (instead of the local population) which, in settings with low background autoimmunity, may result in low test specificity and high false-positive rates [6–8].

In addition, few studies in SSA have reported detailed information on the correlation between islet autoantibody positivity and key metabolic characteristics like optimum markers of pancreatic beta-cell function (either oral insulinogenic index, fasting or 120-minute C-peptide concentration or homeostatic model assessment 2- beta-cell function/HOMA2-%B) and insulin resistance or sensitivity (HOMA2-insulin resistance and QUICKI) [11, 12, 15, 18, 20].

To address these gaps, we undertook the Uganda DIabetes Phenotype (UDIP) study in which we measured three common islet autoantibodies (GADA, IA2A, and ZnT-8A) to screen for pancreatic autoimmunity in patients with recently diagnosed adult-onset diabetes in Uganda. We compared socio-demographic, clinical, and metabolic (including insulin secretion and resistance indices) characteristics between antibody-positive and antibody-negative participants to identify characteristics independently associated with islet autoantibody positivity.

## Materials and methods

### Study sites and duration

The study participants were recruited from outpatient diabetes clinics of seven tertiary public and private not-for-profit (PNFP) mission or church-founded hospitals located in Central and Southwestern Uganda. These particular hospitals serve urban, semi-urban, and rural populations, and most of the patients self-refer to these hospitals for chronic disease management, with a minority being referred from the lower-tier healthcare centres. All patients aged ≥18 years with new-onset and long-standing diabetes are usually managed and followed up at the outpatient diabetes clinics of these tertiary hospitals. About 85% of Ugandans receive medical treatment from public and PNFP hospitals.

For this study, participants that reside in areas legally designated as cities, towns, and municipalities by the Ministry of Lands, Housing, and Urban Development of the Republic of Uganda were classified as an urban population while those living outside the designated cities, towns, and municipalities were classified as a rural population. All participants living in peri-urban areas (within an arbitrary 10-kilometre radius from a city, town, or municipal) were considered an urban population.

The study was carried out from February 2019 to October 2020.

### Study participants

We recruited 534 participants aged ≥18 years with recently diagnosed diabetes (diabetes diagnosed within the preceding three months) of any type from the diabetes outpatient clinics in Uganda. Both treatment naïve and patients on glucose-lowering therapy were included. The diagnosis of diabetes would have been made by clinicians at the different general outpatient clinics based on either fasting blood glucose (FBG) concentration ≥7 mmol/l, random blood glucose concentration ≥11.1 mmol/l with signs and symptoms suggestive of hyperglycaemia, or glycated haemoglobin (HbA1c) concentration ≥6.5% (48 mmol/mol) as recommended by the World Health Organisation guideline on the diagnosis of diabetes [21].

After a diagnosis of diabetes is made, patients are usually then referred to the outpatient diabetes clinics for further management. All recruited study participants were black Africans of Ugandan origin. Pregnant women were excluded. Critically ill patients that required urgent hospitalisation were not immediately recruited at the time of presentation but were eligible if they re-attended the clinic in a stable condition within three months of diagnosis.

### Assessment of socio-demographic, clinical, and biophysical characteristics

All study participants were assessed after an overnight fast of ≥8 hours. Using a pre-tested study questionnaire and standardised study procedures, we collected relevant socio-demographic (age at diagnosis, gender, and residence) and clinical data (history of admission at diagnosis, presence of urine or serum ketones at admission, and diabetes therapies initiated at diagnosis), and undertook biophysical measurements including resting blood pressure and anthropometric measurements (weight, height, waist circumference [WC], hip circumference [HC]—for calculation of body mass index [BMI], waist: hip circumference ratio [WHR] and waist circumference: height ratio [WHtR]). Body composition (total body fat and visceral fat levels) was evaluated using bioimpedance analysis with an OMRON^®^ BF511 body composition monitor.

### Assessment of metabolic characteristics and laboratory measurements

A fasting blood sample was collected to measure blood glucose (FBG), HbA1c, insulin, C-peptide, lipid profile, and the three islet autoantibodies (GADA, ZnT8-A, and IA-2A). This was followed by a 75-gram oral glucose tolerance test (OGTT), with blood samples drawn again 30 and 120 minutes after glucose ingestion to determine the serum glucose, insulin, and C-peptide concentrations at those two time-points. The blood samples were collected in serum separating tubes (SST) II and ethylenediamine tetra-acetic acid (EDTA) tubes and immediately kept at 3°C and room temperature, respectively at each study site before being transported to the clinical chemistry laboratory. Similar temperatures were maintained during transport. On arrival at the clinical chemistry laboratory, the samples were aliquoted and stored at -80°C.

All these laboratory tests were performed at the ISO-certified clinical chemistry laboratory at Medical Research Council/Uganda Virus Research Institute and London School of Hygiene and Tropical Medicine Uganda Research Unit, Entebbe Uganda within three days of sample collection using electro-chemiluminescence immunoassays manufactured by Roche diagnostics Limited, Germany on a Cobas 6000 C-model SN 14H3-15 machine (Hitachi High Technologies Corporation, Tokyo Japan).

Fasting blood glucose concentration was determined quantitatively from plasma using the hexokinase enzymatic principle, with a limit of detection of 0.24–40 mmol/L while HbA1c concentration was determined quantitatively from whole blood by the turbidimetric inhibition immunoassay principle, with a limit of detection of 23–196 mmol/mol (4.2–20.1%). Insulin and C-peptide concentrations were determined quantitatively from serum using the immunoassay sandwich principle, with the limit of detection for insulin and C-peptide of 0.2–1000 u/ml and 0.999–4433 pmol/L, respectively. Lipids were measured quantitatively from serum using the enzymatic colorimetric principle, with the limits of detection for triglycerides, total cholesterol, low-density lipoprotein cholesterol, and high-density lipoprotein cholesterol of 0.1–10 mmol/L, 0.1–20 mmol/L, 0.1–14 mmol/L, and 0.08–3.88 mmol/L, respectively.

Pancreatic autoantibody testing was undertaken using autoantibody ELISA kits from RSR Limited (Cardiff CF14 5DU, UK) on the Dynex DS2 ELISA Robot (Dynex Technologies, Worthing, UK). The lower detection limit of this islet cell autoantibody assay at +2 standard deviations was 1.3 u/mL with an intra-assay and inter-assay precision %CV of 4.4–7.9% and 3.3–5.8%, respectively, and sensitivity and specificity of 94% and 95.6%, respectively [22]. The islet autoantibody testing process also involved a rigorous external laboratory validation exercise with paired samples measured in duplicate for all participants at the Clinical Chemistry and Immunology laboratories, Royal Devon and Exeter NHS Foundation Trust, Exeter United Kingdom.

The online homeostatic model assessment-2 (HOMA2) calculator by the Diabetes Trial Unit of the University of Oxford, Oxford UK was used to calculate the insulin resistance (HOMA2-IR) and the pancreatic beta-cell function (HOMA2-%B) [23]. Pancreatic beta-cell function was also assessed using oral insulinogenic index (IGI) that was calculated using the formula: IGI = difference between the serum insulin concentration at the 30-minute and 0-minute time point/difference between the glucose concentration at the 30-minute and 0-minute time point [24]. The quantitative insulin sensitivity check index (QUICKI) was calculated from fasting serum glucose and insulin concentrations using the online QUICKI calculator [25].

### Definition of islet autoantibody positivity and prevalent diabetes subtypes

Islet autoantibody positivity was confirmed present if GADA, IA-2A, and ZnT8-A levels were >34 U/ml, >58 U/ml, and >67.7 U/ml, respectively. These diagnostic thresholds were obtained after measuring the concentrations of the three islet autoantibodies in archived serum samples of 600 randomly selected healthy Ugandan adults without diabetes (defined as HbA1c <5.5% and random blood glucose <5 mmol/l) that were enrolled in the Medical Research Council/Uganda Virus Research Institute and London School of Hygiene and Tropical Medicine Uganda Research Unit general population cohort. The mean concentration of GADA, IA-2A, and ZnT8-A in this control cohort was 13.4 U/ml, 17.2 U/ml, and 13.3 U/ml, respectively, with the autoantibody concentrations ranged from 2.5 to 1229 U/ml for GADA, 2.6 to 971 U/ml for IA-2A, and 4.2 to 720 U/ml for ZNT8A. The diagnostic thresholds representing the 97.5^th^ percentile (to give a 97.5% specificity) were >38 U/ml, >58 U/ml, and >67.7 U/ml for GADA, IA2A, and ZnT8-A, respectively.

Participants who tested positive and negative for the three islet autoantibodies were classified as having presumed autoimmune diabetes and confirmed type 2 diabetes, respectively.

### Sample size estimation

Using the Leslie Kish formula (1965. Survey Sampling, New York: John Wiley and Sons, Inc.) of sample size calculation, n = Z^2^Pq/d^2^ where n = sample size, Z = 1.96, the normal value corresponding to the 95% confidence interval, d = 3% as the margin of error and a prevalence (P) of GADA positivity in 235 patients with type 2 diabetes in Nigeria of 14% [19], we estimated a sample size of a minimum of 514 participants.

### Statistical analysis

The categorical and continuous variables describing the study participants were expressed as proportions and medians with inter-quartile range (IQR), respectively. The prevalence of positivity for the islet autoantibodies was expressed as frequencies. To assess the socio-demographic, clinical, anthropometric, and metabolic characteristics of participants associated with islet autoantibody positivity, we used the *x*^2^ test for categorical variable comparisons and the Kruskal Wallis test to compare medians for continuous data between the two groups (islet autoantibody-positive and negative participants).

Based on the *x*^2^ and Kruskal Wallis tests, variables that were found to be associated with the islet antibody positivity were then fitted in univariable and multivariable logistic regression models to assess the effect of each of the variables on the main outcome. Logistic regression was used to estimate the effect of each of the potential predictor variables on the main outcome. Univariable logistic regression were fitted to estimate the crude effect of each predictor variable on the outcome. Variables found to have an effect on the outcome were then added to the multivariable logistic regression model one at a time to estimate the effect of each of the predictor variables in the presence of other variables. A new model including all potential predictors identified when investigated one at a time was then fitted. The model was tested for goodness of fit using the Hosmer-Lemeshow test specifying 10 groups.

Odds ratios and 95% confidence intervals from the different models were then obtained and reported. All analyses were done using STATA statistical software version 15 College Station, TX: StataCorp LLC. A p-value <0.05 was considered statistically significant.

### Ethical approval

This study was approved by the Research Ethics Committee of Uganda Virus Research Centre, Entebbe Uganda on 25^th^ May 2018 (GC/127/18/05/650) and the Uganda National Council of Science and Technology on 29^th^ October 2018 (HS 2431). Administrative approval was also obtained from all participating study sites. All enrolled study participants provided written informed consent to participate in the study. For participants who could not read and write, a thumbprint was used to express informed consent in addition to written informed consent offered by an impartial witness representing the illiterate participant.

## Results

### Baseline characteristics of all study participants

The socio-demographic, clinical, anthropometric, and metabolic characteristics of all study participants are summarised in Table 1.

**Table 1:** Socio-demographic, anthropometric, and metabolic characteristics of all study participants and participants with and without islet autoantibody positivity separately.

Characteristic	All study participants (n = 534)	Patients with islet autoantibody positivity (n = 34)	Patients without islet autoantibody positivity (n = 500)	P value
**Socio-demographic and clinical**				
Age at diagnosis, years*				
	48 (39–57)	48 (39–57)	48 (39–58)	1.00
Gender§				
Male	236 (44.2)	19 (55.9)	217 (43.4)	0.16
Female	298 (55.8)	15 (44.1)	283 (56.6)	
Residence§				
Urban	386 (72.4)	16 (47.1)	370 (74.2)	0.005
Rural	145 (27.2)	18 (52.9)	127 (25.5)	
History of admission at diagnosis§				
	220 (41.4)	18 (52.9)	202 (40.6)	0.31
Presence of urine or serum ketones at admission§				
	77 (30.9)	7 (33.3)	70 (30.7)	0.80
Treatment used§ Metformin Sulfonylureas				
	425 (79.6)	24 (70.6)	401 (80.2)	0.18
	199 (37.3)	8 (23.5)	191 (38.2)	0.09
Insulin	153 (28.7)	19 (55.9)	134 (26.8)	<0.001
Systolic blood pressure, mmHg*	126 (115–137)	125 (119–132)	126 (115–137)	1.00
Diastolic blood pressure, mmHg*	84 (77–91)	83 (79–87)	84 (77–91)	0.83
Anthropometry*				
Body mass index, kg/m^2^				
	27.3 (23.5–31.3)	24.9 (21.1–30.4)	27.4 (23.6–31.4)	0.06
WC, cm	95.5 (86.5–104)	90 (80–99)	96 (87–104.8)	0.04
HC, cm	103 (95.5–111)	99 (92–107)	103 (96–111.5)	0.11
WHR	0.92 (0.88–0.96)	0.92 (0.85–0.95)	0.92 (0.88–0.96)	1.00
WHtR	0.59 (0.53–0.65)	0.55 (0.50–0.63)	0.59 (0.53–0.65)	0.03
Total body fat, %	36.3 (26.1–45.1)	31.9 (20.1–39.4)	36.4 (26.5–45.3)	0.24
Visceral fat level	9 (7–12)	8 (5–12)	9 (7–12)	0.18
Metabolic*				
TC, mmol/l	4.0 (3.2–5.0)	3.9 (3.1–4.8)	4.0 (3.3–5.0)	0.50
HDLC, mmol/l	0.9 (0.8–1.2)	0.9 (0.8–1.2)	1.0 (0.7–1.2)	0.79
TGL, mmol/l	1.3 (1.0–1.8)	1.2 (0.8–1.7)	1.3 (1.0–1.8)	0.36
LDLC, mmol/l	2.6 (1.9–3.4)	2.3 (1.9–3.1)	2.6 (1.9–3.4)	0.24
Non-HDLC, mmol/l	3.0 (2.3–3.8)	2.5 (2.1–3.7)	3.0 (2.4–3.8)	0.06
TC/HDLC	4.2 (3.4–5.3)	4.1 (3.4–5.2)	4.2 (3.4–5.3)	0.69
TGL/HDLC	1.4 (1.0–2.2)	1.5 (0.9–2.0)	1.4 (1.0–2.2)	0.84
HbA1c, %	10.4 (7.7–12.5)	11.0 (8.6–12.7)	10.3 (7.7–12.5)	0.46
HbA1c, mmol/mol	90 (61–113)	97 (71–115)	90 (61–113)	0.49
Fasting blood glucose, mmol/l	8.6 (6.3–13.3)	7.7 (6.6–11.3)	8.6 (6.2–13.4)	0.42
Fasting serum insulin, μU/ml	6.0 (3.0–10.5)	6.8 (3.3–10.5)	5.9 (3.0–10.6)	0.48
Fasting serum C-peptide, ng/ml	1.4 (0.8–2.7)	0.9 (0.6–1.8)	1.4 (0.8–2.1)	0.01
30 min blood glucose, mmol/l	13.0 (9.9–18.1)	12.5 (9.9–17)	12.5 (9.9–17.0)	0.71
30 min serum insulin, μU/ml	10.9 (5.4–22.4)	8.4 (4.7–17.0)	11.1 (5.5–22.5)	0.35
30 min C-peptide, ng/ml	2.1 (1.1–3.3)	1.4 (0.9–2.8)	2.1 (1.1–3.3)	0.03
120 min blood glucose, mmol/l	17.4 (12.6–23.3)	19 (15.3–23.0)	17.2 (12.3–23.3)	0.26
120 min serum insulin, μU/ml	13.8 (6.8–27.6)	13.9 (5.8–36.4)	13.7 (6.9–27.1)	0.98
120 min serum C-peptide, ng/ml	2.8 (1.4–4.8)	2.6 (1.1–4.6)	2.8 (1.5–4.8)	0.70
HOMA2-IR	1.22 (0.77–2.03)	1.24 (0.79–2.13)	1.21 (0.77–2.03)	0.90
QUICKI	0.35 (0.31–0.42)	0.34 (0.31–0.42)	0.35 (0.31–0.42)	0.50
HOMA2-%B	43.4 (20.7–77.0)	44.9 (20.2–70.4)	43.1 (20.7–77.6)	0.90
Oral insulinogenic index	1.30 (0.47–3.86)	0.67 (0.31–2.01)	1.31 (0.47–3.86)	0.21

§Results are presented as numbers and proportions

*Results are presented as median (inter-quartile range/IQR)

HbA1c-Glycated haemoglobin, HC-Hip circumference, HDLC-High dense lipoprotein cholesterol, HOMA2-%B-Homeostatic model assessment-beta cell function, HOMA2-IR- Homeostatic model assessment- insulin resistance, LDLC-Low dense lipoprotein cholesterol, TC-Total cholesterol, TGL-Triglycerides, QUICKI- quantitative insulin sensitivity check index, WC-waist circumference, WHR-Waist: hip circumference, WHtR- waist circumference: height ratio.

The median (IQR) age at diagnosis, BMI, HbA1c and fasting C-peptide for all the participants was 48 (39–57) years, 27.3 (23.5–31.3) kg/m^2^, 10.4 (7.7–12.5) % (90 [61–113] mmol/mol), and 1.4 (0.8–2.7) ng/ml, respectively. 56% of all study participants were females and about 81% (n = 432) of the participants were enrolled in the study within two months of diagnosis. Approximately 30% of participants had a history of the presence of ketones in serum or urine at the time of admission. Insulin therapy was initiated immediately following diagnosis in about 29% of participants.

### Prevalence of islet autoantibody positivity

**Fig 1:**
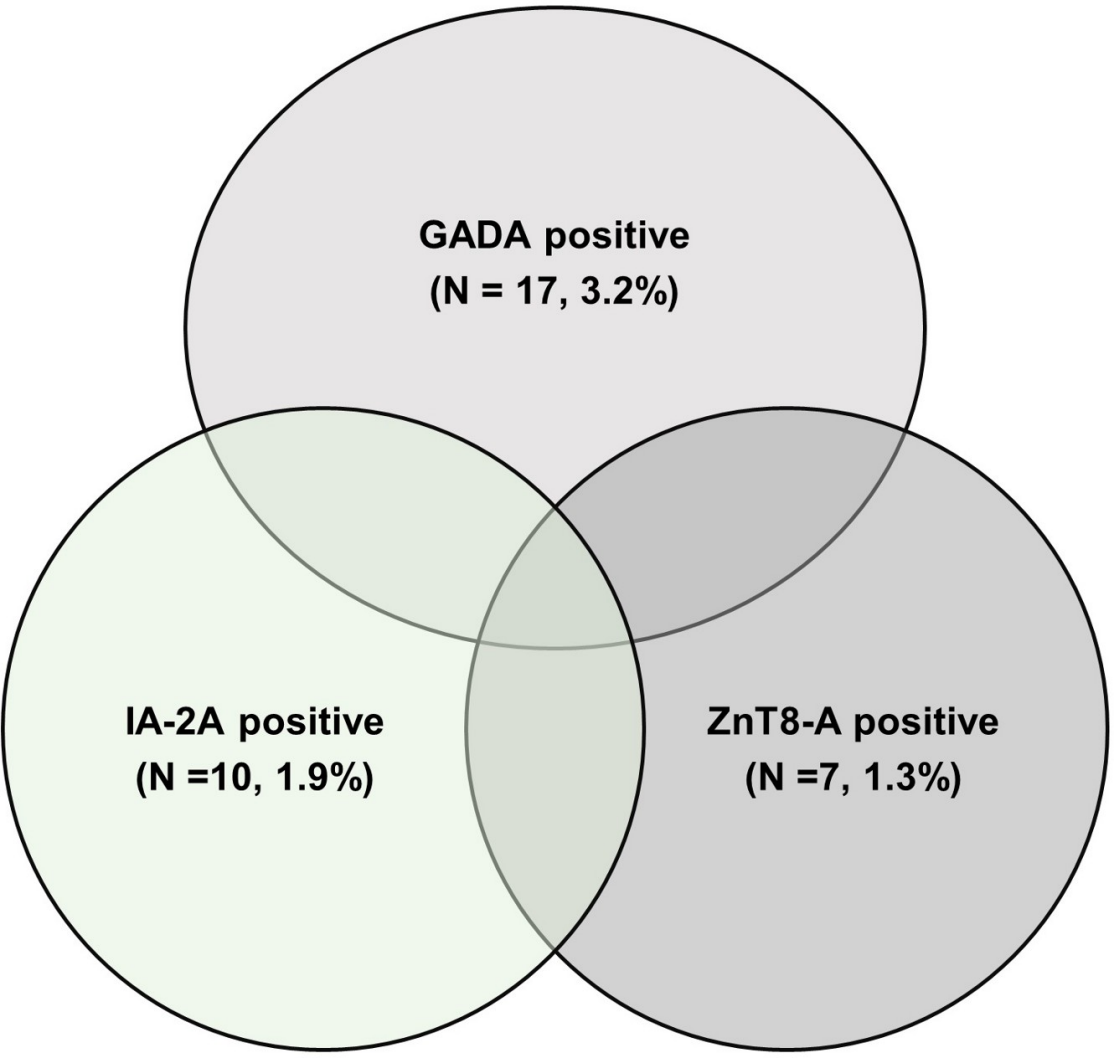
The pattern of islet autoantibody positivity in participants positive for islet autoantibodies (n = 34). GADA-Autoantibody against glutamic acid decarboxylase-65, IA-2A- Antibody against tyrosine phosphatase, ZnT8-A: autoantibody against zinc transporter 8.

Thirty four participants (6.4%) were positive for at least one of the three islet autoantibodies. GADA, IA-2A, and ZnT8-A were positive in 17 (3.2%), 10 (1.9%), and 7 (1.3%) patients, respectively. Of the 34 participants that were positive for islet autoantibodies, only four (11.8%) were positive for >1 autoantibody, all of whom had GADA and ZnT8-A. Fig 1- [Pattern of islet autoantibody positivity].

### Socio-demographic, clinical, anthropometric, and metabolic characterisation of participants with islet autoantibody positivity

The socio-demographic, clinical, anthropometric, and metabolic characteristics of participants with and without islet autoantibody positivity are summarised in Table 1.

Participants who were positive for islet autoantibodies were more likely to live in a rural area (n = 18, 52.9% vs n = 127, 25.5%, p = 0.005) and to have a lower median WC (90 [80–99] cm vs 96 [87–104.8], p = 0.04) and WHtR (0.55 [0.50–0.63] vs 0.59 [0.53–0.65], p = 0.03), compared to those who were negative. In addition, a greater proportion of participants with positive islet autoantibodies were immediately started on insulin therapy following diagnosis (n = 19, 55.9% vs n = 134, 26.8%, p<0.001). No differences in age at diagnosis were noted between the two groups.

Differences in BMI and body composition measures between those with and without islet autoantibody positivity were not statistically significant (BMI-24.9 [21.1–30.4] vs 27.4 [23.6–31.4] kg/m^2^, p = 0.06, total body fat- 31.9 [20.1–39.4] vs 36.4 [26.5–45.4] %, p = 0.24, and visceral fat level- 8 [5–12] vs 9 [7–12], p = 0.18).

Participants who had islet autoantibodies had significantly lower median fasting C-peptide (0.9 [0.6–1.8] vs 1.4 [0.8–2.1] ng/ml, p = 0.02) and 30-minute fasting C-peptide levels (1.4 [0.9–2.8] vs 2.1 [1.1–3.3], p = 0.03). No statistically significant differences were observed with other measures of pancreatic beta-cell function (HOMA2-%B (44.9 [20.2–70.4] vs 43.1 [20.7–77.6], p = 0.90) and oral IGI (0.67 [0.31–2.01 vs 1.31 [0.47–3.86], p = 0.21) and insulin sensitivity (QUICKI- 0.34 [0.31–0.42] vs 0.35 [0.31–0.42], p = 0.50). Similarly, no significant differences were seen in circulating levels of metabolic markers such as FBG, HbA1c, lipid profile, and HOMA2-IR.

### Predictors of islet autoantibody positivity on multivariate analysis

Table 2 summarises the clinical and metabolic characteristics that were independently associated with islet autoantibody positivity.

**Table 2:** Predictors of islet autoantibody positivity on multivariate analysis.

Characteristic	AOR (95% CI)	P-value
Age at diagnosis (years)	0.98 (0.96–1.01)	0.26
Rural residence	3.62 (1.68–7.80)	0.001
Initiation of insulin therapy at diagnosis	3.61 (1.67–7.83)	0.001
Waist circumference (cm)	0.98 (0.96–1.01)	0.23
Fasting C-peptide (ng/ml)	1.01 (0.70–1.46)	0.95

AOR- Adjusted odds ratio, CI- confidence intervals

On multivariate analysis, living in a rural area (OR 3.62, 95%CI 1.68–7.80, p = 0.001) and being initiated on insulin therapy at the time of diagnosis (OR 3.61, 95% CI 1.67–7.83, p = 0.001) were independently associated with islet autoantibody positivity.

## Discussion

To the best of our knowledge, this is the first study in SSA to simultaneously screen for islet autoantibody positivity (defined using local population-derived diagnostic thresholds) based on testing three islet autoantibodies in adult patients with recently diagnosed diabetes using a high performing islet autoantibody assay in the international Islet Autoantibody Standardisation Program with concurrent rigorous external laboratory validation.

Our study shows that islet autoantibody positivity is relatively infrequent in adult patients with recently diagnosed diabetes in Uganda. The prevalence of islet autoantibody positivity in our cohort was close to the expected autoantibody-positive rate in a population without autoimmune diabetes. With the use of 97.5% specificity test thresholds, we would expect 2.5% of those without autoimmune diabetes to test positive for each test [6–8]. This, therefore, is consistent with a very low prevalence of autoimmune diabetes in this study population and suggests that routine testing for islet autoantibodies in adult Ugandan patients with recently diagnosed diabetes would result in many false positives. The rates of false-positive results would further increase if the lower manufacturer’s cut-offs were used to define islet autoantibody positivity.

Islet autoantibody positivity is thought to be common in European populations with adult-onset diabetes (4.5–9.7%) with GADA positivity rates being significantly higher than those of IA-2A and ZnT8-A (4.5–11.1% vs 0.2–2.3%, respectively) [26–29]. In one study performed in a selected adult Czech population with LADA, confirmed maturity-onset diabetes of the young, and healthy controls, a high prevalence of ZnT8-A positivity of 23.7% was noted in 59 participants with study-defined LADA. All of these participants were positive for GADA and IA-2A [30].

Lower prevalence rates based on the positivity of either two or all the three islet autoantibodies (GADA, IA-2A, and ZnT8-A) have been reported in other populations, such as in the Middle East (2.8% based on GADA and IA-2A positivity) [31] and Asia (1.5%-8.6% based on GADA, IA-2A, and ZnT8-A positivity) [32–36]. Similar to findings of European population-based studies, GADA positivity has been reported to be more prevalent than IA-2A and ZnT8-A positivity in Asians with phenotypic type 2 diabetes [34–36].

Generally, few studies have screened for islet autoantibody positivity in unselected adult populations with diabetes (where specific types of diabetes are not excluded) using more than one islet autoantibody. Most studies in SSA have screened for only GADA positivity and have reported higher prevalence rates compared to what we observed in our study (3.2%).

Two studies conducted in Kenya and Tanzania in adult patients with apparent type 2 diabetes reported almost similar prevalence of GADA positivity of 5.7% and 5.3%, respectively [11, 12]. Screening for IA-2A positivity in the latter study increased the prevalence of islet autoantibody positivity to 7.3% [12]. Higher prevalence levels of GADA positivity in adult-onset diabetes have also been reported in Madagascar (12%) [15] and in West African populations in Nigeria (10.5–14%) [13, 14, 19] and Ghana (8.9–14.3%) [16, 17].

The reason for this apparent difference in the prevalence of GADA positivity noted in our study and the highlighted studies above is likely to be the varying study definition of GADA positivity. The majority of those studies used manufacturer’s cut-off points to define positivity. In contrast, we found that the manufacturer’s cut-off point was considerably lower than the local population-derived cut-off, which we used in our study, and using the manufacturer’s cut-offs would increase the frequency of false-positive tests, leading to inaccurate estimates of the prevalence of islet autoantibody positivity [6–8]. This need for using appropriate population-defined references ranges to define islet autoantibody positivity was recently also demonstrated in a large study of type 1 diabetes in Ethiopia, where a GADA assay and threshold well-established in European populations had poor specificity, testing positive in 8.5% of those without diabetes [37].

On univariate analysis, islet antibody-positive individuals were more likely to live in a rural area, to be initiated on insulin therapy at diagnosis, and exhibited lower measures of obesity (WC and WHtR) and pancreatic beta-cell function (fasting and postprandial C-peptide). These findings are consistent with what has been observed in participants with islet autoantibody positivity in other populations [6, 26, 27, 38, 39]. In addition to having lower markers of obesity, a finding of lower fasting and postprandial C-peptide concentrations in islet autoantibody-positive participants as shown in our study is an indicator of reduced pancreatic beta-cell function which is linked to progressive autoimmune-mediated damage of the pancreatic beta-cells [40]. A high prevalence of pancreatic beta-cell dysfunction based on a lower fasting C-peptide concentration in participants with islet autoantibody positivity has been widely documented in several similar studies [39, 41–43]. No association between islet autoantibody positivity and insulin resistance was observed in this study.

Living in a rural area and being initiated on insulin therapy at diagnosis were noted to be independently associated with islet autoantibody positivity in this study population on multivariate analysis. The observation of an association between living in a rural area and islet autoantibody positivity is of special interest and is supported by previous studies. For example, in a study in Ghana, islet autoantibody positivity was seen more commonly in rural areas compared to urban areas (14.3% Vs 8.9%) [16]. Furthermore, one of the highest prevalence levels of islet autoantibody positivity in Africa (28%) has been reported from a rural semi-arid famine-prone area in Ethiopia [18]. The underlying mechanisms that increase the likelihood of pancreatic autoimmunity in rural areas are unclear, but chronic malnutrition has been implicated [44, 45].

Initiation on insulin therapy at diagnosis as an independently associated factor of islet autoantibody positivity in this study population signifies pancreatic beta-cell dysfunction which is a predictor of early initiation of insulin therapy in islet autoantibody-positive participants in several studies [26, 27, 41, 42, 46].

In European and Asian population-based studies, patients with adult-onset diabetes and positive for islet autoantibodies are significantly younger at diagnosis and have lower BMI, blood pressure, and a more favourable lipid profile, when compared to those with type 2 diabetes [26–28, 39]. In contrast, data from our study and most of the studies in African patients have not demonstrated these differences between patients with and without islet autoantibody positivity [14, 15, 17, 19]. While this may partly be due to the low numbers of patients positive for islet autoantibodies, several studies and clinical observations have suggested that Africans appear to develop type 2 diabetes at a young age and lower levels of BMI, which might influence these relationships [10].

### Strengths and limitations

This study recruited a cohort of adult patients with recently diagnosed diabetes within three months of diagnosis to minimise the decline in rates of islet autoantibody positivity that occurs with increasing diabetes duration [47, 48]. Screening for islet autoantibody positivity was based on testing three common autoantibodies using diagnostic thresholds derived from an appropriate healthy adult Ugandan population without diabetes, as widely recommended, hence ensuring a high test specificity [6–8]. The study also used one of the highest performing islet autoantibody assays in the international Islet Autoantibody Standardisation Program [49] with extensive validation performed on paired samples in an external laboratory (Royal Devon and Exeter NHS Foundation Trust, Exeter UK), to ensure robust results. The study also assessed an additional number of metabolic characteristics, especially measurements of pancreatic beta-cell function, insulin resistance, and sensitivity.

Limitations of our study include recruitment from only specialist diabetes clinics of the tertiary hospitals which potentially introduces a selection bias and might result in reporting a higher burden of islet autoantibody positivity in patients with adult-onset diabetes, as milder cases may be more likely to be managed without attending a specialist clinic. However, the majority of patients in Uganda are seen in these clinics in tertiary hospitals for the management of chronic diseases, with very limited provision of diabetes care in lower-tier district hospitals. It is also possible that our delayed recruitment of those requiring admission (with recruitment only offered on re-attending the diabetes clinic) could have reduced the prevalence of islet autoantibody positivity in this cohort, leading to an underestimate of the prevalence. We, however, managed to recruit most of these patients following discharge from the hospital at their subsequent clinical review.

Because of the small number of participants with islet autoantibody positivity, the study had limited power to detect differences in some clinical characteristics in this population. Lastly, because we performed multiple tests, we cannot rule out chance findings.

## Conclusion

The prevalence of islet autoantibody positivity in this study population of adults with recently diagnosed diabetes in Uganda was relatively low, suggesting that pancreatic autoimmunity is a rare cause of adult-onset diabetes in the Ugandan population. Due to the low rates of islet autoantibody positivity in this study population, routine testing of islet autoantibodies would have limited clinical significance in Uganda and would likely result in many false-positive results, especially if the lower manufacturer’s cut-offs are used to define autoantibody positivity. The study finding of an association between living in a rural area and islet autoantibody positivity is of unique interest and warrants further investigation.

## Supporting information

S1 Data (XLSX)
